# Notes and News

**Published:** 1905-09-02

**Authors:** 


### Notes and News.

A general hospital is in course of erection in the city of Mexico, at a cost of 2,191,950 dollars.

A lady, Miss F. Sabin, has been appointed Associate Professor of Anatomy in the Johns Hopkins University.

A very fine hospital (according to the Consul-General's report) has just been completed at Punta Arenas, a flourishing town in the most southerly department of Chile.

Mr. Edgar Schuster, the Francis Galton Scholar in National "Eugenics," will publish a report on his research into the inheritance of disease. It will appear in the first volume of the publications of the Eugenics Record Office.

It is a long time since anything like an epidemic of cholera has appeared in Europe. Now we learn that since August 16th cases of cholera have been occurring continuously in various localities in West Prussia, through which the Vistula runs. The outbreak is mainly amongst Russian lumbermen who are bringing timber rafts to the coast. The river authorities are placing infected persons in quarantine and taking every precaution to check the spread of the disease.

A good deal of local interest is excited at St. Leonards and Hastings by the proposal to remove the General Hospital from the Parade, probably to a higher and freer situation. The present institution was designed by Messrs. Young and Hall, and was opened in 1887. It cost about £20,000. If a really good and sufficient site is available the proposal to remove the hospital from its present noisy and cramped position has our full sympathy. The site at present occupied by this hospital must be of considerable value.

The reports are still very bad from New Orleans. Leeville is the worst centre of yellow fever, and here 170 cases have been reported.

Improvements have recently been made in the Civil Hospital at St. Helena. It is now connected with the new system of drainage and water supply installed in Jamestown, and a verandah has been erected communicating with the wards. There were 112 admissions to the hospital during last year, and three deaths.

The hospital at Gambia has been enlarged and thoroughly repaired, and is now one of the best on the West Coast of Africa. A new wing has been erected for female patients. The total number of admissions to the hospital in 1904 was 666. There are, besides the senior medical officer, three assistant medical officers for the colony.

A new process of producing flour from wheat is on exhibition at 93 and 94 Upper Thames Street. It is the invention of Mr. Apostoloff, who claims for it that a yield of 85 per cent. of flour can be obtained against 68 to 72 per cent. by the ordinary method of milling. It is also claimed for it that the flour extracted by his process is more nourishing and wholesome.

Very few of the public know the cost of an epidemic to the ratepayer. Isolation is essential and the most economical procedure in the long run, but the expense of a fever patient is, nevertheless, a very heavy burden to the community. A Richmond inhabitant has analysed the cost incurred by the district in this direction, and he states that each patient costs £5 5s. a week, or about £37 for the duration of his illness.

Col. David Bruce, of the Royal Army Medical Corps, delivered a most interesting lecture on sleeping sickness at Pietermaritzburg last week. He explained that sleeping sickness, the infection of which had been traced to the Glossina morsitans fly, was spreading wherever there existed the particular fly to convey it. He pointed out that there was a danger of it even spreading to India, where a somewhat similar disease already exists amongst horses.

The proceedings of the Plymouth Guardians in regard to the question of a new workhouse infirmary, is very characteristic. A good many meetings have already been held without any decision resulting. The majority of the guardians will not even admit that the present provision for the sick is in any way faulty. One guardian claims they are "no worse off than they were ten or twelve years ago," whilst another proposes that a labour colony should be formed for the employment of the more able-bodied inmates of the workhouse, and that the space vacated by them could be devoted to the sick and to the accommodation of nurses. A guardian also remarked that something ought to be done if only to justify the board before the public. As the matter stands at present plans for a new wing are to be submitted to the Local Government Board.

